# Seasonal extreme temperatures and short-term fine particulate matter increases child respiratory hospitalizations in a sparsely populated region of the intermountain western United States

**DOI:** 10.21203/rs.3.rs-3438033/v1

**Published:** 2023-10-17

**Authors:** Erin L. Landguth, Jonathon Knudson, Jon Graham, Ava Orr, Emily A. Coyle, Paul Smith, Erin O. Semmens, Curtis Noonan

**Affiliations:** University of Montana; University of Montana; University of Montana; University of Montana; University of Montana; University of Montana; University of Montana; University of Montana

**Keywords:** asthma, case-crossover design, environmental health, hospital discharge data, lag effects, lower respiratory tract infections, Montana, PM2.5, rural, upper respiratory tract infections, wildfire

## Abstract

**Background:**

Western Montana, USA, experiences complex air pollution patterns with predominant exposure sources from summer wildfire smoke and winter wood smoke. In addition, climate change related temperatures events are becoming more extreme and expected to contribute to increases in hospital admissions for a range of health outcomes. Few studies have evaluated these exposures (air pollution and temperature) that often occur simultaneously and may act synergistically on health.

**Methods:**

We explored short-term exposure to air pollution on childhood respiratory health outcomes and how extreme temperature or seasonal period modify the risk of air pollution-associated hospitalizations. The main outcome measure included all respiratory-related hospital admissions for three categories: asthma, lower respiratory tract infections (LRTI), and upper respiratory tract infections (URTI) across western Montana for all individuals aged 0–17 from 2017–2020. We used a time-stratified, case-crossover analysis and distributed lag models to identify sensitive exposure windows of fine particulate matter (PM_2.5_) lagged from 0 (same-day) to 15 prior-days modified by temperature or season.

**Results:**

Short-term exposure increases of 1 μg/m^3^ in PM_2.5_ were associated with elevated odds of all three respiratory hospital admission categories. PM_2.5_ was associated with the largest increased odds of hospitalizations for asthma at lag 7–13 days [1.87(1.17–2.97)], for LRTI at lag 6–12 days [2.18(1.20–3.97)], and for URTI at a cumulative lag of 13 days [1.29(1.07–1.57)]. The impact of PM_2.5_ varied by temperature and season for each respiratory outcome scenario. For asthma, PM_2.5_ was associated most strongly during colder temperatures [3.11(1.40–6.89)] and the winter season [3.26(1.07–9.95)]. Also in colder temperatures, PM_2.5_ was associated with increased odds of LRTI hospitalization [2.61(1.15–5.94)], but no seasonal effect was observed. Finally, 13 days of cumulative PM_2.5_ prior to admissions date was associated with the greatest increased odds of URTI hospitalization during summer days [3.35(1.85–6.04)] and hotter temperatures [1.71(1.31–2.22)].

**Conclusions:**

Children’s respiratory-related hospital admissions were associated with short-term exposure to PM_2.5_. PM_2.5_ associations with asthma and LRTI hospitalizations were strongest during cold periods, whereas associations with URTI were largest during hot periods.

**Classification:**

environmental public health, fine particulate matter air pollution, respiratory infections

## BACKGROUND

Less than 1% of the world experiences daily concentrations of fine particulate matter air pollution (< 2.5 μm in aerodynamic diameter; PM_2.5_) that is less than the recommended daily safe levels (Yu et al. 2023). The daily safe thresholds and related policies have been set based on rigorously designed epidemiological cohort and time series studies (e.g., Pope et al. 1995, Pope et al. 2009), confirmed through rigorous re-analysis and subsequent studies over the last several decades (Krewski et al. 2004, Krewski et al. 2009, WHO 2013, Atkinson et al. 2014). PM_2.5_ affects many health outcomes, but of interest in this study, the role of PM_2.5_ in respiratory health is well known for a range of conditions, including upper respiratory tract infections (URTI; e.g., croup; Dylag et al. 2018, laryngitis; Cheng et al. 2021, influenza; Meng et al. 2021, COVID-19; Kim et al. 2022), lower respiratory tract infections (LRTI; e.g., bronchitis; Kurmi et al. 2010, bronchiolitis; Karr et al. 2006, pneumonia; Ngung et al. 2017), and chronic disorders (e.g., chronic obstructive pulmonary disease; Sunyer et al. 2006, asthma; Guarnieri and Balmes 2014, lung cancer; Fajersztajn et al. 2013). Associative impact studies overwhelmingly corroborate a correlative link between respiratory health outcomes and exposure to air pollutants (e.g., US EPA 2009, Kim and Kabir 2015, Liu et al. 2017), as well as delayed exposures through both short-term (i.e., 1 day-1 month; e.g., Galan et al. 2003, Wong et al. 2008, Xie et al. 2019) or long-term timeframes (1 month-1 year; e.g., Orr et al. 2020, Landguth et al. 2020). Inhaling PM_2.5_ can produce inflammation and oxidation stress, triggering cellular damage and increasing the risk of respiratory disease (Black et al. 2017).

Ambient PM_2.5_ air pollution, particularly in urban and higher-income country settings, has been significantly reduced over the last 40 years (McClure and Jaffe 2018, Ford et al. 2018). However, in some areas of the world, and specifically for our rural and intermountain study setting of Montana, USA, exposure to PM_2.5_ continues to increase due to residential wood combustion for heat in the winter season and wildfire smoke events during the summer (or wildfire) season. In the 2022 State of the Air report (American Lung Association 2022), Montana received failing grades for eight counties based on the number of unhealthy and hazardous air-quality days due to severe wildfires and use of residential wood stoves. Regarding wood stoves, Montana ranks second in the USA in the proportion of households that heat with wood fuel (7.4% compared to 1.7% in the USA; ACS 2020). Chemical Mass Balance source apportionment studies have shown that residential wood stoves are the largest source of ambient PM_2.5_ during the winter months (55.5–82%; Ward 2009, Ward and Lange 2010, Ward 2016). Studies evaluating the health impacts associated with residential sources of PM_2.5_ are limited and often suffer from challenges related to sparse populations and uncertain generalizability (Noonan and Balmes 2010, Sigsgaard et al. 2014).

The second air quality threat in the mountain west region is smoke from nearby and distant wildfires, a PM_2.5_ source that is projected to worsen with climate change (Ford et al. 2018, O’Dell et al. 2019). A growing body of literature is focused on the health effects of PM_2.5_ specifically derived from wildfire smoke. Health impacts from wildfire smoke exposures range from irritation of the eyes and respiratory tract to respiratory morbidity, with growing evidence supporting an association with all-cause mortality (Reid et al. 2016). In particular, hospitalizations and emergency department visits related to respiratory infections and preexisting conditions, such as asthma and COPD, are consistently elevated during and shortly following wildfire events (Reid et al. 2016, Cascio 2018). Several factors complicate the evaluation of wildfire exposures and healthcare usage on health outcomes. These include uncertainty in lag effects and potential non-linear response curves that may indicate lower healthcare utilization during extremely high wildfire smoke events, perhaps mediated through behavior changes that are not at play in urban settings where the moderately elevated PM exposures are less recognizable or notable by community members (Gould et al. 2023).

In parallel, global exposure to extreme temperatures has grown and is expected to worsen with climate change. Extreme temperature events, both cold and hot, are known to be associated with excess mortality and increased hospital admissions for a range of health outcomes (USGCRP 2016, Gasparrini et al. 2017). Hotter days in the summer will cause increased levels of illness and death by compromising the body’s ability to regulate its temperature, or by exacerbating health problems. Cold temperatures in the winter can cause blood vessels to constrict, heightening cardiovascular issues, and irritating the airways triggering respiratory problems and lower immunity. Specifically focusing on the respiratory-related health categories in this study, literature for temperature-associated respiratory health effects are mixed with respect to hot versus cold temperature extremes and depending on the respiratory categories studied. For example, it is well known that cold (and dry) conditions can increase the survival rate of influenza viruses and enhance viral spread (e.g., Lowen et al. 2007). A recent review concluded that both extreme heat and cold could significantly increase the risk of asthma (Han et al. 2022). Sheerens et al. (2022) showed that higher temperatures may worsen dyspnea, while colder temperature may trigger cough and phlegm symptoms among COPD patients.

Multiple rigorous studies of have observed impacts on health of PM_2.5_ and temperature, but have considered increases in these exposures separately; however, these exposures often occur simultaneously and may act synergistically on health. The potential for interactive effects based on these two climate-relevant factors is important as current population risk estimates and corresponding policy recommendations are based largely on epidemiological studies quantifying the effects of PM_2.5_ and temperature considered in isolation. A systematic review of several studies, almost entirely in urban populations, indicate sufficient findings of moderate quality to support synergistic effects for temperature and air pollution (Anenberg et al. 2020), although such evidence for pediatric respiratory outcomes is extremely limited (Winquist et al. 2014). Assessment of these questions in rural communities also is limited, but a recent case crossover study in California for all age cardiorespiratory hospitalization showed strong evidence for a synergistic effect between wildfire specific PM_2.5_ and extreme heat (Chen et al. 2023). Although the authors did not specifically evaluate rurality, they found weaker interactions between temperature and air pollution in counties with higher education attainment, health insurance coverage, income, and automobile ownership, possibly due to greater capacity to reduce harmful exposures in individuals of higher socioeconomic status.

For the study presented here, we evaluated associations between short-term or delayed fine particulate matter (PM_2.5_) on three respiratory health outcomes assessed at the individual level. We additionally assessed modification of these associations by temperature and season. We focused on a rural and sparsely populated service area in western Montana, USA, from 2017–2021. This area of the inter-Rocky Mountains is experiencing more frequent exceedance of daily air quality standards in the summer due to increases in wildfire smoke events with the largest source of ambient PM_2.5_ in the winter due to residential wood stoves. This area of the northern hemisphere also has more winter cold months than summer warm months, though average annual temperatures are rising.

## METHODS

All analyses were performed with R software (version 4.2; R Development Core Team) including the ‘lme4’ (Bates et al. 2015), ‘Tidyverse’ (Wickham et al. 2019), and ‘biostat3’ (Tillander et al. 2022) packages.

### Study Area, Population, and Respiratory Health Outcomes

The study protocol was approved by the Institutional Review Board (IRB) at the University of Montana. Initial study approval was obtained by the University of Montana-Missoula Institutional Review Board on 6 July 2021 (#97–21). Health data were previously collected administrative data; thus informed consent requirements did not apply.

#### Study area

**Figure 1:**
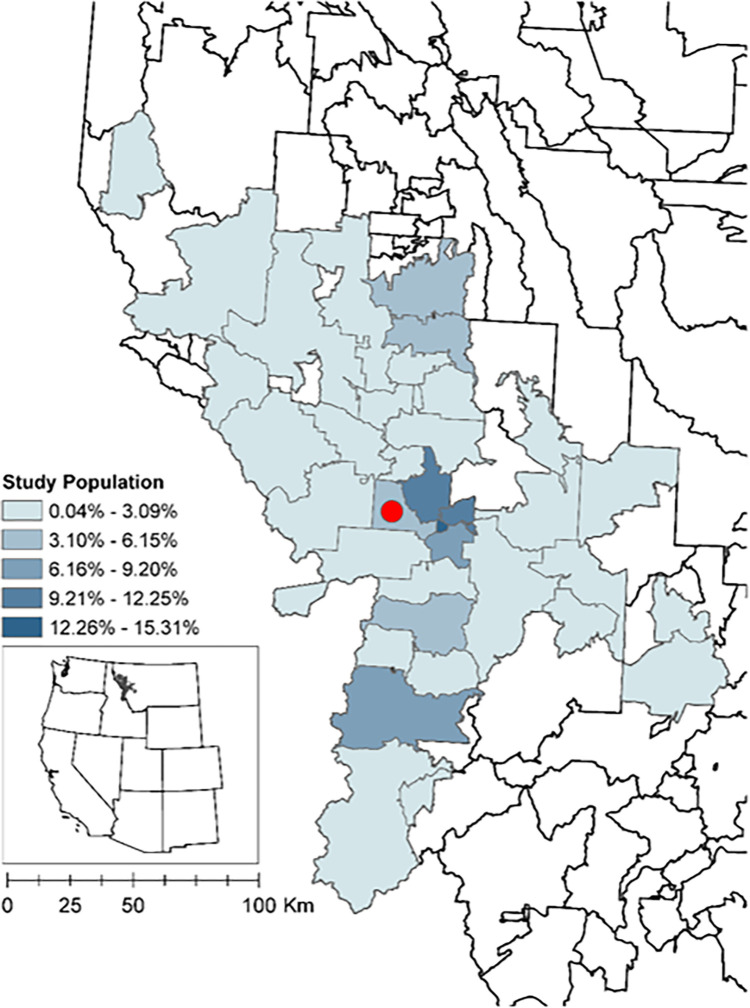
Study Area Population. 45 ZCTAs in western Montana included in study area symbolized by percent total population. Hospital location (ZCTA = 59804) represented by red circle.

For our study, we are focused on western Montana, USA (Fig. 1). The study area covers 45 out of 361 Montana Zip Code Tabulation Areas (ZCTA) across 8 of the 56 counties (Deer Lodge, Granite, Lake, Mineral, Missoula, Powell, Ravalli, and Sanders). The total population within this area was approximately 233,657 in 2020 that includes one small city (Missoula, population total = 73,948) surrounded by sparsely populated areas (US Census Bureau 2020). According to the US Census Bureau’s definition of rurality, this study area is defined as having 72.3% of the population living in rural areas. For context, the US has 19.3% of the population living in rural areas (Ratcliffe et al. 2016). This region of the inter-Rocky Mountains is experiencing more frequent exceedance of daily air quality standards in the summer months (particularly in July–September; Landguth et al. 2020) due to increases in wildfire smoke events. The largest source of ambient PM_2.5_ in the winter is due to residential wood stoves (Ward 2009). At this northern hemisphere latitude (45–49°N), Montana experiences more winter cold months (3.4 on average) than summer warm months (2.8 on average) (Frankson et al. 2022). Annual average temperatures, including daily minimums, maximums, and averages, have risen across Montana; between 1950 and 2015, with increases ranging from 1.1–1.7°C (Whitlock et al. 2017). Both wildfire smoke events and warming temperature periods are expected to increase in number across Montana through the 21st century.

##### Hospital admissions data:

Hospital data were collected from December 2017–September 2021 for one hospital that predominantly serves Missoula County in western Montana, United States, with 14,814 respiratory-related records. These data included nine sources of admissions type: clinic, inpatient, emergency, observation, outreach clinic, preadmission outpatient clinic, professional services, provider clinic, and telemedicine clinic. We removed records with residential addresses that fell outside western Montana, resulting in a reduced spatial sample of 14,071 records. We further right-censored the date range beginning on 13 March 2020 to remove the influence of the COVID-19 pandemic on result findings (final n = 10,133). Data included individuals aged 0–17 with a respiratory-coded infection (see Case definitions for health outcomes below and Table 1). A strictly protected health protocol was implemented through data use agreements between the records provider and the University of Montana, where personal identifiers were removed, and residential addresses were geocoded and geomasked. The individual-level data and corresponding spatiotemporal daily PM_2.5_ exposure values were used in case-crossover analyses (see Case-crossover design and analysis).

#### Case definitions for health outcomes

**Figure 2:**
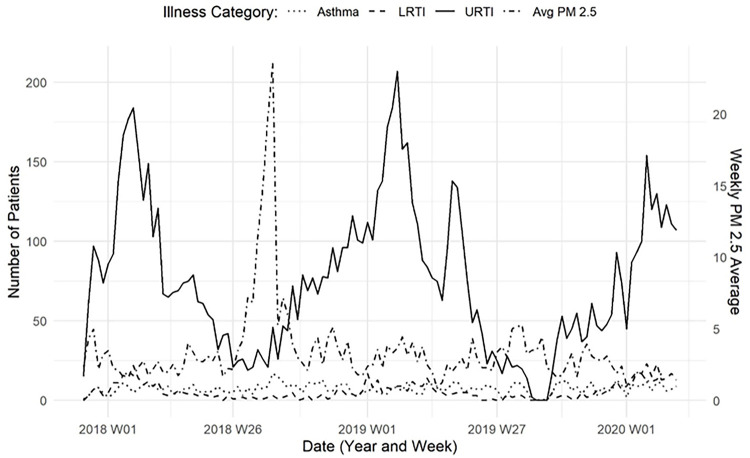
Respiratory Hospital Admissions and PM_2.5_. Related hospital visits for asthma (dotted line), lower respiratory tract infections (LRTI–dashed line), and upper respiratory tract infections (URTI–solid line), by week, for western Montana residents, aged 0–17. Average PM_2.5_, shown in dot-dashed line for the entire study area, for comparison.

For this study, cases related to upper respiratory tract infections (URTI), lower respiratory tract infections (LRTI), and asthma were first identified using the International Classification of Diseases,10th Revision, Clinical Modification diagnosis codes (ICD-10-CM) and sorted following the case definitions of the Armed Forces Health Surveillance Center (AFHSC 2015) (Table 1). We further identified and split case definitions based on each infection’s upper and lower airway occurrences. Records were classified by condition when a related diagnosis code of interest was found in the primary diagnosis field (first-listed) or any secondary diagnosis field (1–8). Records were selected once for each associated category. For example, records with more than one URTI code were only counted once for the URTI category. If a record had codes for URTI, LRTI, and asthma, the record was counted once in each of the three categories. Hospital data in the study period and area are shown as total counts for each respiratory category in Fig. 2, along with average weekly PM2.5.

### Exposure, Outcome, and Other Explanatory Variables of Interest

All environmental exposure data used in modeling are summarized in Table 2.

#### PM2.5 exposure assessment

The daily time-series dataset of PM_2.5_ surface concentrations was previously developed, and details are reported elsewhere (Swanson et al. (2022). Briefly, these data were produced from air quality station observations, satellite data, and meteorological data to produce daily 1-km resolution surface PM_2.5_ concentration estimates to explore health outcome impacts of PM_2.5_ across spatiotemporal domains specific to the rural and intermountain western USA. We extracted daily PM_2.5_ measurements for each case event address location on date of hospital visit for the case-crossover modeling, along with PM_2.5_ at the address location for reference days (see Case-crossover design and analysis).

##### Delayed PM 2.5 exposure effects:

To identify sensitive windows of PM2.5 exposure and test the effects of short-term and delayed PM2.5 effects on respiratory health, we investigated the effect of PM2.5 from 0 to 15 days before the admission date with 3 variations of PM2.5 lags: (i) single day – single day PM2.5 lags include just the day previous to the case event, (ii) cumulative days – cumulative days of PM_2.5_ include cumulative PM_2.5_ for all *k* days prior to the case event day, for *k* = 1, …, 15, and (iii) weekly average – weekly average PM2.5 lags include rolling averages of PM2.5 over 1-week periods prior to the case event, including 0–6, 1–7, …, 9–15 days prior.

#### Temperature

We included daily maximum temperature modeled by gridMET (Abatzoglou 2013) extracted to each individual location, date of admission and corresponding reference days for the case-crossover model. We used the 15th, 50th, and 85th percentiles of temperature (cutoffs for colder = −0.7 °C, median = 6.2 °C, and hotter = 20.7 °C) to estimate the effect of the interaction of PM_2.5_ and temperature on the three respiratory outcomes.

#### Season

In Montana, PM_2.5_ levels spike during summer season due to the primary source of wildfire smoke and during the winter season due to the primary source of wood smoke (Ward 2009). We therefore included a categorical season predictor that is assumed to be associated with the exposure of interest and potentially also associated with the respiratory health outcomes of interest. We included a northern hemisphere season as a categorical variable that included summer (June–August), fall (September–November), winter (December–February), and spring (March–May). The model also employed an interaction between season and PM2.5 to allow for differential effects by season of PM2.5 on the health outcomes. In addition, we included a three-way interaction between season, PM_2.5_, and temperature to assess potential different interactive effects of PM_2.5_ and temperature by season.

### Statistical modeling

#### Case-crossover design and analysis

Introduced in environmental health studies by Maclure (1991), case-crossover designs compare an individual’s (case) exposure immediately prior to or during the defining case event with that same individual’s exposure at different reference times. This method is attractive because it compares individuals with themselves and controls for time invariant confounders and secular trends by design. Since the seminal Maclure (1991) study, several variations on choosing control days to minimize biases have emerged, and convergence to a time-stratified case-crossover design has evolved as the recommended approach for minimizing sources of bias (see Wu et al. 2021 for review).

Thus, we evaluated the synergistic effect of temperature extremes specific to a season and the 3 types of PM_2.5_ lag predictors on the risk of each respiratory infection outcome (asthma, LRTI, or URTI) using a time-stratified case-crossover design widely used in studies of short-term environmental health exposures (e.g., Talbot et al. 2014; Wei et al. 2019). We created case-crossover datasets for each respiratory outcome, with paired case events and either 3 or 4 controls. The case event day was defined as the date of hospital admission. We then identified matched control days as the same weekdays from other weeks of the same month and year in the same geocoded location of residence (i.e., of the same person). Matching by day of the week controlled for potential confounding by factors that vary within a week (e.g., weekend/weekday differences in hospital admission rates). We selected control days both before and after the case day to minimize bias from long-term time trends in PM_2.5_ (Levy et al. 2001). Time-invariant factors between the case event day and each control day, such as age, sex, race, socioeconomic status, and other short timeframe changing health behaviors, were assumed unlikely to change (Lu and Zeger 2007).

For each respiratory binary response (asthma, LRTI, or URTI) and corresponding case-crossover dataset, we applied a conditional logistic model to estimate the odds ratio of the effect of PM_2.5_ at 3 temperature levels (colder, median, hotter) and within 4 seasons (Chen et al. 2023). The conditional logistic model accounts for the case/control matching by comparing the exposures of each matched case and control, and calculating a weighted average across all matched sets (Di et al. 2017). We estimated odds ratios and corresponding 95% confidence intervals for relative odds of hospitalization for each 1 μg/m^3^ increase in PM_2.5_, within each temperature/season combination, and for each of the 3 respiratory outcomes. An odds ratio larger than one indicates the exposure variable increases odds of hospitalization. Modification of the effects of PM_2.5_ on each respiratory health outcome by temperature was assessed by including multiplicative interaction terms for PM_2.5_ and temperature (colder, median, hotter). We employed a similar approach to assess interactions between PM_2.5_ and season. The three-way interaction terms for PM_2.5_xTemperaturexSeason were not included due to model instability. To account for the effects of temperature or season, appropriate linear combinations of coefficients were utilized using the ‘biostat3’ R package (Tillander et al. 2022).

#### Distributed lag modeling

Distributed lag modeling is a flexible approach that allows one to simultaneously model exposure-response and lag-response relationships (Gasparrini 2014). Using the case-crossover modeling framework described above, distributed lag models were used to determine sensitive windows of PM_2.5_ exposure on risk of respiratory hospital admission. These models used lagged PM_2.5_ concentration as the exposure variable along with PM2.5xTemperature, and PM2.5xSeason interactions.

## RESULTS

In summary, we analyzed respiratory hospital admission data for a sparsely populated region in western Montana, USA. During the study period (1 December 2017–13 March 2020), we observed 10,133 respiratory admissions among 8,128 unique patients, including 794 asthma, 638 LRTI, and 8,392 URTI. Figure 2 illustrates the weekly case counts and seasonal patterns across the time period studied. Modeled daily PM_2.5_ concentrations within our study area and period ranged from 0.45 to 40.40 μg / m^3^ with a mean of 3.64 (standard deviation (SD) = 3.64) μg / m^3^. Mean values for the year 2018 were 4.51 (SD = 5.56, median = 2.83, interquartile range (IQR) = 2.66) μg / m^3^ and for the year 2019 were 3.15 (SD = 2.44, median = 2.54, IQR = 2.02) μg / m^3^. Daily mean PM_2.5_ exceeded the United States Environmental Protection Agency 24-hr standard 35 μg / m^3^ on 6 of 821 days (0.7%). Notably, all 6 of these days that exceeded the daily standard occurred during August 2018 when a prolonged air pollution event was experienced in the area due to smoke transport from extensive wildfire activity in the western US and Canada. In what follows, we report results for each respiratory outcome (asthma, LRTI, URTI). Consistent results occurred for the distributed lag models that used cumulative days of PM_2.5_ and weekly average of PM_2.5_ with the strongest relationships between PM2.5 and the respiratory outcomes occurring around 7–3 day lag. Single day PM_2.5_ values are shown only in supplementary material.

### Asthma:

The increase in the risk of hospitalization for asthma associated with each 1 μg / m^3^ increase in PM_2.5_ modified by temperature or season can be found in **Figures A.1**-**A.3** and Table 3A. For the main effect of PM_2.5_, we observed associations with asthma hospitalizations at weekly average lag 7–13 [OR = 1.87, 95% CI: (1.17–2.97); **Fig. A.1C**]. In colder temperatures and during the same lag of 7–13 days prior to a healthcare visit, a 1 μg / m^3^ increase in PM_2.5_ was associated with 3.11-fold greater odds of asthma [95% CI: (1.40–6.89); **Fig. A.2C**]. Accumulated PM_2.5_ (0–13 days) resulted in consistent findings with patterns of a stronger odds for PM_2.5_ at colder versus median temperatures [OR = 2.43 95% CI: (1.05–5.64) and OR = 1.97 95% CI: (1.03–3.76), respectively; **Fig. A.2B**]. Finally, in the winter season and during the same lag of 7–13 days prior to an admission, a 1 μg / m^3^ increase in PM2.5 was associated with 3.26-fold increase odds of asthma hospitalizations [95% CI: (1.07–9.95); **Fig. A.3C**]. No significant PM_2.5_ effects on asthma during hotter temperatures or other seasons (besides winter) were observed.

### LRTI:

The increase in the risk of hospitalization for LRTI associated with each 1 μg / m^3^ increase in PM_2.5_ modified by temperature or season can be found in **Figures A.4**-**A.6** and Table 3B. LRTI had the lowest sample size of the respiratory health outcome categories studied here (n = 638), resulting in unstable odds estimates for some model groups. However, of the models that had larger samples sizes, the PM_2.5_ only model was associated with LRTI hospitalizations at weekly average lag 6–12 days [OR = 2.18 95% CI: (1.20–3.97); **Fig. A.4C**]. In colder temperatures and during the same lag of 6–12 days prior to a healthcare visit, a 1 μg / m^3^ increase in PM_2.5_ was associated with 2.61-fold greater odds of LRTI hospitalization [95% CI: (1.15–5.94); **Fig. S5C**]. No significant associations for PM_2.5_ effects on LRTI hospitalization during hotter temperatures or any season were seen, although case frequencies for some of these model groups were small (e.g., n = 34 in the summer season).

### URTI:

**Figure 3:**
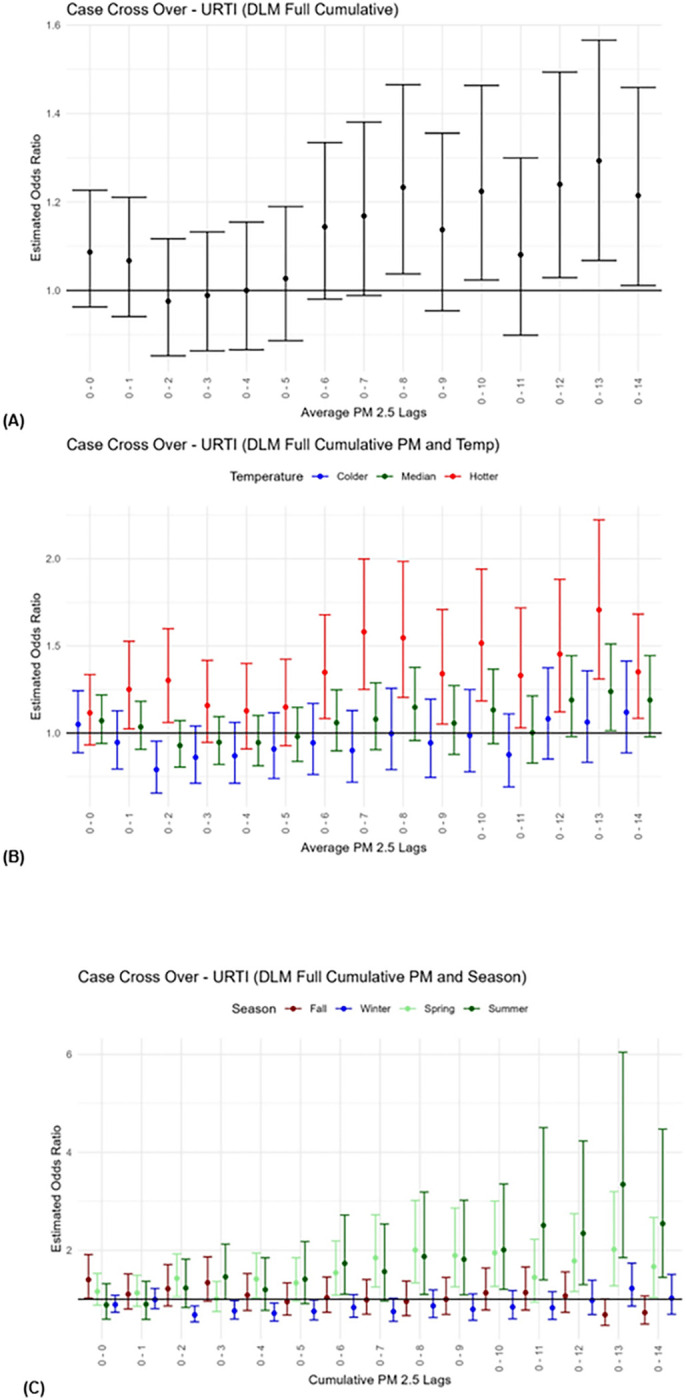
Children’s Upper Respiratory Tract Infection Risk for Hospital Admissions with Short-Term Exposure to PM2.5. Case cross-over results for distributed lag models of PM2.5 for delays in cumulative days for (A) the Main PM2.5 model–only PM2.5, (B) the Temperature model–PM2.5xTemperature displayed for three levels of hotter (red), median (green) and colder (blue) temperatures, and (C) the Season model–PM_2.5_xSeason displayed for the four levels of Fall (maroon), Winter (blue), Spring (light green), and Summer (green).

The increase in the risk of hospitalization for URTI associated with each 1 μg / m^3^ increase in PM_2.5_ modified by temperature or season can be found in Fig. 3, **Figures A.7**-**A.9**, and Table 3C. For the main effect PM_2.5_ only model, highest odds estimates for URTI hospitalizations were associated with 1 μg / m^3^ increase in cumulative PM_2.5_ in the 13 days prior to a healthcare visit [OR = 2.43 95% CI: (1.05–5.64)]. In general, for cumulative lag models the odds estimates remained elevated beyond 0–5 days. The higher frequency of URTI outcomes, relative to asthma and LRTI outcomes, allowed for consistent findings of interactive effects by temperature and season and indicated that PM_2.5_ effects were present in hotter rather than colder conditions. At cumulative lag (0–13 days), summer days yielded the highest PM_2.5_ association with 3.35-fold increased odds of URTI hospitalization [95% CI: (1.85–6.04)]. Similar patterns were observed for hotter days and summer/spring seasons across multiple lag periods.

## DISCUSSION

We found short-term increases in PM_2.5_ air pollution were positively associated with children’s respiratory related hospital visits for a patient population in western Montana, USA. These effects were found for categories of respiratory related visits of asthma (peak odds at lag of 7–13 days), LRTI (peak odds at a lag of 6–12 days), and URTI (peak odds after 13 accumulated days). These results are consistent with findings from past studies. While, in general, consistency of findings implies an association between increased respiratory risk and increased PM_2.5_, the length of the observed lag effect does vary (as highlighted below). These links between increased respiratory risk and increased short-term PM_2.5_ are well established. That PM_2.5_ impacts vary by temperature and season is a novel contribution of our study. We showed that PM_2.5_ associations with asthma and LRTI hospitalization are strongest during colder temperatures. By contrast PM_2.5_ effects on URTI hospitalization were elevated during hotter temperatures and during the summer season.

### Asthma and PM_2.5_ exposure:

Numerous studies link air pollution to asthma. Several reviews have highlighted this connection, specifically for exacerbating existing asthma, but also with an increase of new-onset asthma (Guarnieri et al. 2014, Tiotiu et al. 2020). A recent meta-analysis of 84 studies including children, adults, or both found that outdoor air pollutants were associated with an increased risk of asthma exacerbations at lag 0–1 days (Huang et al 2021). The study also conducted age-based subgroup analyses of children (0–14) and adults (>14) and found children with asthma were more susceptible to outdoor air pollution (Huang et al 2021). However, various other time-series studies using air pollutants have observed a lag effect with varying results from 0–5 days (Ostro et al. 2001, Lee et al. 2006, Halonen et al. 2008, Khalili et al. 2018, Lu et al. 2020, Puvvula et al. 2022) to 6–7 days (Chien et al 2018, Dabrowiecki et al. 2022). In general, lag effects past 7 days are typically not observed, though Gu et al. (2004) found a lag of 7–10 days. Our study, using the DLM, places the PM_2.5_ associated increased risk in children’s asthma events at a weekly average lag of 7–13 days.

### Asthma, extreme temperatures, and seasonal effects:

Our study indicated the highest risk for asthma hospitalization at PM_2.5_ weekly average lag 7–13 days during colder temperatures or during the winter season. Of course, above the 45 ^0^N parallel, these two factors for colder temperatures and winter season are no doubt, conflated. However, very cold and dry or very hot and humid climate conditions have been shown to exacerbate asthma conditions (Cong et al. 2017, Fang et al. 2021, Lam et al. 2016). An animal model demonstrated that high and low temperatures can aggravate airway inflammation in mice suggesting that asthmatics are more at-risk during exposures to high and low temperature extremes (Deng et al. 2020). A recent review found that extreme cold exposures were associated with an increased risk of asthma by 19.77% (Han et al. 2023). Seasonal effects on asthma are inconclusive most likely because a range of temperature conditions have been shown to affect asthma risk. However, increased asthma risk has been observed in fall and winter seasons (Teach et al. 2015).

### LRTI and PM2.5 exposure:

In this study, LRTI encounters for children increased with elevated PM2.5 and peaked at a weekly average lag of 6–12 days Studies on this category of respiratory infections or specific infections within this category (e.g., bronchitis or pneumonia) vary in their findings. To discuss a few, numbers of acute lower respiratory infections for young children in Utah, USA, were found to increase after 1 week of increased PM_2.5_ and peak after 3 weeks of an increased exposure (Horne et al. 2018), while a similar study and results from Korea found acute lower respiratory infection hospitalizations to be associated with an increase in the 7-day running average of PM_2.5_ (Oh et al. 2021). Zhu et al. (2017) did not find a significant effect of short-term PM_2.5_ on childhood lower respiratory diseases in China, but did observe the effect with other air pollutants (PM_10_, NO_2_, and SO_2_). In New York, USA, increases in PM2.5 from the previous 7 days were found to be associated with hospital admissions for culture-negative pneumonia and bacterial pneumonia (Croft et al. 2018). To further illustrate variability in results, a meta-analysis review of short-term exposure to PM_2.5_ and pneumonia-related hospitalizations found variable results across study populations, where elderly subgroups showed an increased risk ratio with unclear lag effects and younger patients did not have a significant increase in visits (Kim et al. 2020).

### LRTI, extreme temperatures, and seasonal effects:

Our study showed the highest risk estimates for LRTI as a function of PM_2.5_ during colder temperatures but insufficient sample size to assess any seasonal PM_2.5_ mediated effects. These results are in line with past studies showing LRTI to be a significant cause of hospitalizations, morbidity, and mortality worldwide with seasonal climate factors being associated with a higher probability of infection (Fares et al. 2013). For example, Alvaro-Meca et al. (2022) found that LRTI hospital admissions were more frequent during lower temperatures. And Mäkinen et al. (2008) demonstrated that cold temperatures were associated with increased occurrences of LRTI and a decrease in temperature preceded the onset of infections.

### URTI and PM_2.5_ exposure:

URTI have also been extensively studied and linked to air pollutants. Here, we found a positive association between PM_2.5_ and children’s URTI hospitalizations with a peak response at 13 days of accumulated PM_2.5_ prior to an admission. As with asthma and LRTI, past research has shown that study population, study region, methodology, and type of upper respiratory tract infection can produce variations in the length of the delayed short-term effects. For example, in Beijing, China, a positive association between PM_2.5_ and increased influenza cases suggested a 1–2 month delayed response (Liang et al. 2014). In Hefei, China, increasing concentrates of most all pollutants at lag days 3–6 were associated with increased URTI in children aged 0–14 years (Li et al. 2018), while in Suzhou City, China, PM_2.5_ showed a significant association with these infections in children under 3 years old with a lag of 3 weeks (Zhang et al. 2019). In Kenya, a 2-week delayed response in children’s URTI from PM_2.5_ exposure was observed (Larson et al. 2022). In Poland, moderate exposure to air pollution over 12 weeks was associated with an increased risk of URTI in children aged 3–12 years (Rataiczak et al. 2021).

### URTI, extreme temperatures, and seasonal effects:

Our study showed relationships between increased risk of URTI hospitalization and delayed and elevated PM_2.5_, temperature, and season. Elevated levels of PM_2.5_ accumulated across 13 days during hotter temperatures or during the summer season yielded the highest risk of children’s URTI hospitalization. In general, URTI are thought to be more common in colder temperatures because colder exposure impairs nasal antiviral immunity (Eccles 2002, Huang et al. 2023). Viral infectious diseases affecting the upper tract way, such as influenza, have strong seasonal effects in winter temperate regions and are associated with colder temperatures (Price et al. 2019). However, not all URTI spike in winter months in northern temperate sites, and others, like enterovirus and parainfluenza virus, can occur in summer months and respiratory syncytial virus can occur earlier than influenza in fall months (Lam et al. 2019). Rhinoviruses and adenoviruses can circulate throughout the year with occasional peaks in autumn and winter for rhinoviruses and early spring for adenoviruses (Jacobs et al. 2013, Dela Cruz et al. 2019). In summary, most respiratory viruses follow a seasonal pattern but not all URTI are viruses, and some factors can increase the incidence of URTI, like mass crowding (Ahmed et al. 2006), and, as was observed in this study, air pollution.

### Limitations:

Air pollution case-crossover studies are not without limitations. First, a note on sample size. These data cover 821 days with an average of 12.8 events per day among all three outcomes (1.06, 0.84, and 10.9 events for asthma, LRTI, and URTI, respectively). It has been suggested in simulation studies of pollution effects, that thousands of observation days with an average of tens of events per day are needed (Winquist et al. 2012). We observed instability in some of our estimates, particularly for those models with low sample sizes (LRTI and PM_2.5_xSeason). Second, unmeasured time-variant factors might have provided additional confounding influence and could possibly impact estimates (Bateson and Schwartz, 1999, 2001). To the degree that these factors occur at the individual level, e.g., immunity or vaccination status, the impact is likely to be negligible given the case-crossover design. Third, error in diagnostic coding is possible. Some cases may not be accurately categorized, and it is possible that such coding errors could be differential with respect to season. Finally, the assessment of exposure could be subject to measurement (and modeling) error, especially in a rural, sparsely populated study area with only a limited number of fixed air quality monitors contributing to the estimates of PM_2.5_ (Swanson et al. 2022). However, we expect this error would have results in attenuated effect estimates (Wu et al. 2019).

## CONCLUSIONS

Western Montana, USA, is a sparsely populated region of the inter-Rocky Mountains with complex air pollution patterns. This region is experiencing more frequent exceedance of daily air quality standards due to increases in wildfire smoke events during their summer/wildfire season months. However, the region also experiences elevated levels of PM_2.5_ during winter months from wood stove use with complex mountain meteorology and inversion effects. Here, we explored short-term PM_2.5_ effects on three childhood respiratory health outcomes (asthma, LRTI, and URTI) and how other factors, such as extreme temperature or seasonal period, modify the risk of air pollution-associated hospitalizations. We found associations between elevated PM_2.5_ exposures and hospital visits for all respiratory categories. We found interaction effects with extreme temperatures and during high impacted PM_2.5_ seasons. We found increased risk for asthma and LRTI associated with elevated levels of PM_2.5_ in colder temperatures, while increased risk for URTI associated with elevated levels of PM_2.5_ in hotter temperatures or the summer season. Communities in the western US will experience increases in morbidity and mortality related to higher frequency of extreme temperature and wildfire events (Whitlock et al. 2017). At present, policy and public health messaging related to air pollution and extreme temperatures flow through different agency pathways. For example, extreme cold and heat advisories often occur in advance based on local National Weather Service forecasting, and air quality advisories often occur in real-time according to EPA-based Air Quality Index measures. Communities at risk of wildfire smoke exposures and extreme temperature events need locally-informed guidance, integrated strategies that address these compound risks, and communication approaches that include local knowledge and trusted sources. Local communities will be increasingly burdened with developing and sustaining strategies for adaptation and resilience to climate change, but we lack rigorous and reproducible models for such strategies, particularly as applicable to rural communities in the mountain west that additionally suffer from limited infrastructure that can be leveraged for mitigation.

## Figures and Tables

**Table 1:** Case Definitions for Respiratory Infections. ICD-10-CM diagnosis codes for upper respiratory tract infections (URTI), lower respiratory tract infections (LRTI), and asthma.

URTI	J00, J01, J01.0, J01.00, J01.01, J01.1, J01.10, J01.11, J01.2, J01.20, J01.21, J01.3, J01.30, J01.31, J01.4, J01.40, J01.41, J01.8, J01.80, J01.81, J01.9, J01.90, J01.91, J02.0, J02.8, J02.9, J03.00, J03.01, J03.80, J03.9, J03.90, J03.91, J04, J04.0, J04.1, J04.10, J04.11, J04.2, J04.3, J04.30, J04.31, J05, J05.0, J05.1, J05.10, J05.11, J06, J06.0, J06.9, J09.X2, J09.X3, J09.X9, J10, J10.0, J10.00, J10.01, J10.08, J10.1, J10.2, J10.8, J10.81, J10.82, J10.83, J10.89, J11, J11.0, J11.00, J11.08, J11.1, J11.2, J11.8, J11.81, J11.82, J11.83, J11.89, J21.0, J21.8, J21.9, H65, H66, H66.9
LRTI	J20, J20.0, J20.1, J20.2, J20.3, J20.4, J20.5, J20.6, J20.7, J20.8, J20.9, J21, J21.0, J21.1, J21.8, J21.9, J09.X1, J09.X2, A37, A37.00, A22.1, A37.01, A37.10, A37.11, A37.80, A37.81, A37.90, A37.91, A48.1, B25.0, J12, J12.0, J12.1, J12.2, J12.3, J12.8, J12.81, J12.82, J12.89, J12.9, J13, J14, J15, J15.0, J15.01, J15.1, J15.2, J15.20, J15.21, J15.211, J15.212, J15.29, J15.3, J15.4, J15.5, J15.6, J15.7, J15.8, J15.9, J16, J16.0, J16.8, J17, J18, J18.0, J18.1, J18.2, J18.8, J18.9, J18, J21, J20.9, R05.1, R05.2
Asthma	J44.0, J44.1, J44.9, J45, J45.2, J45.20, J45.21, J45.22, J45.3, J45.30, J45.31, J45.32, J45.4, J45.40, J45.41, J45.42, J45.5, J45.50, J45.51, J45.52, J45.9, J45.90, J45.901, J45.902, J45.909, J45.99, J45.990, J45.991

**Table 2:** Explanatory Variable Descriptions. A summary of variables used in modeling is provided. Interactions between these variables were also considered.

Variable	Measure Description and Usage	Reference
PM_2.5_	Daily 1-km gridded surface PM_2.5_ estimates were the primary exposure of interest. We extracted PM_2.5_ values at each case event address on date of admission, all 15 days prior to the date of case admission for investigating short-term exposure effects, and referent days for the case-cross over design (details provided in text).	Swanson et al. 2022
Temperature	Daily 4-km gridded gridMET data were used to extract surface maximum temperature at each case event address on date of admission and corresponding referent days.	Abatzoglou 2013
Season	At each case event day and corresponding referent days, a categorical variable to indicate the season was used for summer (June-Aug), fall (Sep-Nov), winter (Dec-Feb), and spring (Mar-May).	-

**Table 3:** Modification of the effect of PM_2.5_ exposure on respiratory health by temperature or season. The largest relative odds ratios (OR) and corresponding 95% confidence intervals (CI) with P-values (Pval) are displayed here to estimate the increase in odds of hospitalization for each 1 μg/m^3^ increase in PM_2.5_ at the given lagged cumulative days or weekly average for each temperature/season combination, and for each of the 3 respiratory outcomes: (A) asthma, (B) lower respiratory tract infections (LRTI), and (C) upper respiratory tract infections (URTI). For each respiratory health outcome condition, the three models are presented for PM_2.5_, PM_2.5_xTemperature (grouped by Colder, Median, and Hotter), and PM_2.5_xSeason (grouped by Fall, Winter, Spring, and Summer). **Bolded** values indicate an OR with 95% CI that occurred above 1.0. UN indicates unstable estimates. n indicates the sample size for model and group where available, noting that temperature was interacted as a continuous variable and doesn’t define groups.

(A) Asthma								
Model	Group	Cumulative Days			Weekly Average			n
		Lag	OR	Pval	Lag	OR	P	
PM_2.5_	-	0–13	1.46 (0.85–2.51)	0.168	**7–13**	**1.87 (1.17–2.97)**	0.008	794
PM_2.5_xTemp	Colder	**0–13**	**2.43 (1.05–5.64)**	0.038	**7–13**	**3.11 (1.40–6.89)**	0.005	-
	Median	**0–13**	**1.97 (1.03–3.76)**	0.041	**7–13**	**2.44 (1.35–4.39)**	0.003	-
	Hotter	0–12	1.46 (0.76–2.80)	0.471	7–13	1.46 (0.85–2.53)	0.174	-
PM_2.5_xSeason	Fall	0–4	0.90 (0.37–2.44)	0.843	8–14	1.75 (0.75–4.08)	0.197	191
	Winter	0–13	1.28 (0.29–5.76)	0.612	**7–13**	**3.26 (1.07–9.95)**	0.038	247
	Spring	0–2	2.69 (0.94–7.67)	0.064	8–14	1.47 (0.41–5.22)	0.552	190
	Summer	0–13	2.00 (0.67–5.94)	0.693	3–9	2.13 (0.81–5.63)	0.127	166
**(B) LRTI**								
Model	Group		Cumulative Days			Weekly Average		N
		Lag	OR	Pval	Lag	OR	P	
PM_2.5_	-	0–12	2.03 (0.96–4.30)	0.066	**6–12**	**2.18 (1.20–3.97)**	0.011	638
PM_2.5_xTemperature	Colder	0–12	1.84 (0.73–4.65)	0.195	**6–12**	**2.61 (1.15–5.94)**	0.022	-
	Median	0–12	1.97 (0.91–4.27)	0.087	**6–12**	**2.35 (1.26–4.39)**	0.007	-
	Hotter	0–10	2.35 (0.79–6.98)	0.124	4–10	2.26 (0.78–6.55)	0.134	-
PM_2.5_xSeason	Fall	0–12	1.46 (0.19–11.4)	0.716	4–10	1.31 (0.24–7.29)	0.758	61
	Winter	0–13	1.64 (0.36–5.09)	0.460	8–14	1.68 (0.69–4.12)	0.253	374
	Spring	0–2	1.39 (0.42–4.61)	0.591	6–12	2.07 (0.55–7.75)	0.280	169
	Summer	UN	UN	UN	UN	UN	UN	34
**(C) URTI**								
Model	Group		Cumulative Days			Weekly Average		N
		Lag	OR	Pval	Lag	OR	P	
PM_2.5_	-	**0–13**	**1.29 (1.07–1.57)**	0.008	**4–10**	**1.24 (1.06–1.45)**	0.009	8,392
PM_2.5_xTemperature	Colder	0–14	1.12 (0.89–1.41)	0.345	**8–14**	**1.24 (1.02–1.52)**	0.032	-
	Median	**0–13**	**1.24 (1.01–1.51)**	0.036	**6–12**	**1.21 (1.03–1.43)**	0.022	-
	Hotter	**0–13**	**1.71 (1.31–2.22)**	<0.001	**4–10**	**1.31 (1.03–1.67)**	0.031	-
PM_2.5_xSeason	Fall	**0–0**	**1.40 (1.02–1.91)**	0.035	6–12	1.12 (0.82–1.55)	0.474	1,266
	Winter	0–13	1.22 (0.86–1.74)	0.259	**7–13**	**1.46 (1.13–1.91)**	0.004	4,224
	Spring	**0–13**	**2.02 (1.27–3.20)**	0.003	**4–10**	**1.92 (1.32–2.81)**	<0.001	2,254
	Summer	**0–13**	**3.35 (1.85–6.04)**	<0.001	**5–11**	**2.10 (1.28–3.43)**	0.003	648

## Abbreviations

LRTI: lower respiratory tract infections
MT: Montana, USA
PM2.5: fine particulate matter air pollution (<2.5 μm in aerodynamic diameter)
URTI: upper respiratory tract infections
